# New simple and quick method to analyze serum variant transthyretins: direct MALDI method for the screening of hereditary transthyretin amyloidosis

**DOI:** 10.1186/s13023-019-1100-y

**Published:** 2019-05-27

**Authors:** Toshiya Nomura, Mitsuharu Ueda, Masayoshi Tasaki, Yohei Misumi, Teruaki Masuda, Yasuteru Inoue, Yukimoto Tsuda, Masamitsu Okada, Takahiro Okazaki, Kyosuke Kanenawa, Aito Isoguchi, Makoto Nakamura, Konen Obayashi, Satoru Shinriki, Hirotaka Matsui, Taro Yamashita, Yukio Ando

**Affiliations:** 1 Department of Neurology, Graduate School of Medical Sciences, Kumamoto University, 1-1-1 Honjo, Kumamoto, 860-0811 Japan; 2 Department of Morphological and Physiological Sciences, Graduate School of Health Sciences, Kumamoto University, 4-24-1 Kuhonji, Kumamoto, 862-0976 Japan; 3 Department of Molecular Laboratory Medicine, Graduate School of Medical Sciences, Kumamoto University, 1-1-1 Honjo, Kumamoto, 860-0811 Japan

**Keywords:** Hereditary transthyretin amyloidosis, Variant transthyretin, Mass spectrometry

## Abstract

**Background:**

Hereditary transthyretin amyloidosis (ATTRv amyloidosis) is caused by a variant transthyretin (TTR), which is a serum protein secreted by the liver. Mass spectrometry (MS) is a useful tool that can detect variant TTRs in serum samples from patients with ATTRv amyloidosis. We previously reported several mass spectrometric methods to detect variant TTRs in serum samples. Those methods require cumbersome immunoprecipitation with anti-TTR antibodies and significant time to analyze the variant TTRs. In our study here, we developed a new simple and quick method to detect variant TTRs in serum samples by means of matrix-assisted laser desorption-ionization time-of-flight (MALDI-TOF) MS without immunoprecipitation (direct MALDI).

**Methods:**

By using direct MALDI, we analyzed 288 serum samples obtained from patients who were clinically suspected having amyloidosis to investigate the usefulness of this direct MALDI method to detect variant TTRs in serum samples.

**Results:**

The method completed the process within 30 min. We successfully identified variant TTRs in serum samples from patients, except for a few patients with TTR Glu61Lys and Glu89Gln mutations because of the small mass shift of those variant TTRs from wild-type TTR. We also found that the mass shifts of variant TTRs measured by direct MALDI corresponded to theoretical mass changes.

**Conclusion:**

Our results suggest that the direct MALDI method is useful for the screening of ATTRv amyloidosis.

## Background

Hereditary transthyretin (TTR) amyloidosis (ATTRv amyloidosis), which is caused by mutations in the *TTR* gene, is an inherited systemic disorder characterized by extracellular amyloid deposits. Patients with ATTRv amyloidosis develop systemic symptoms such as sensorimotor neuropathy, autonomic dysfunction, cardiomyopathy, gastrointestinal dysfunction, renal failure, and ocular disorders [1]. To date, more than 140 different mutations in the *TTR* gene have been reported, most of which have been associated with ATTRv amyloidosis. Of the pathogenic TTR mutations, Val30Met is most frequently found worldwide [2, 3].

TTR is a plasma protein, which is mainly synthesized in the liver, and acts as a transporter of thyroxine and retinol-binding protein with vitamin A. In the bloodstream, TTR forms a homotetramer with a dimer-of-dimers configuration. TTR mutations cause destabilization of TTR tetramers, which is believed to be a crucial step in formation of TTR amyloid [4]. Regarding this mutation, late-onset patients from non-endemic areas show distinctive clinical features from early-onset patients from conventional endemic foci [5]. Patients with ATTRv amyloidosis, particularly late-onset cases, tend to be initially misdiagnosed as having other diseases [6].

Mass spectrometry (MS) is a powerful tool that can detect small molecular changes in proteins. Because the molecular masses of variant TTRs with amino acid exchanges differ from that of wild-type (WT) TTR, we developed diagnostic methods to detect variant TTRs in serum samples by means of several different mass spectrometric analyses such as matrix-assisted laser desorption-ionization time-of-flight (MALDI-TOF) MS with immunoprecipitated (IP) serum TTR (IP-MALDI) [7–9], electrospray ionization (ESI) MS with IP serum TTR (IP-ESI) [10–12], and surface-enhanced laser desorption/ionization time-of-flight (SELDI-TOF) MS ProteinChip system [13, 14]. Although those methods are valuable for screening ATTRv amyloidosis and double-checking TTR variants in addition to genetic testing of the *TTR* gene, they require significant time to analyze variant TTRs, and they also sometimes fail to detect TTRs because of technical difficulties related to the cumbersome immunoprecipitation with anti-TTR antibodies.

Here, we developed a new simple and reliable mass spectrometric method for ATTRv amyloidosis screening in which we can directly detect variant TTRs in serum samples without pre-purification by using MALDI-TOF MS (direct MALDI).

## Methods

### Patients

Between April 2015 and March 2017, we examined 288 serum samples obtained from patients who were clinically suspected of having amyloidosis. Table 1 provides detailed information about serum samples obtained from 42 patients with ATTRv amyloidosis.

**Table 1:** Variant TTRs detected in 42 serum samples obtained from 42 patients with ATTRv amyloidosis by means of the direct MALDI method

Patient number	TTR mutation	Detection of variant TTR peaks	Theoretical mass changes (*m/z*)	Measured mass changes (*m/z*)
1	Val30Met	+	+ 32.06	+ 31.95
2	Val30Met	+	+ 32.06	+ 31.62
3	Val30Met	+	+ 32.06	+ 31.58
4	Val30Met	+	+ 32.06	+ 31.30
5	Val30Met	+	+ 32.06	+ 32.08
6	Val30Met	+	+ 32.06	+ 31.21
7	Val30Met	+	+ 32.06	+ 32.51
8	Val30Met	+	+ 32.06	+ 31.97
9	Val30Met	+	+ 32.06	+ 31.20
10	Val30Met	+	+ 32.06	+ 31.53
11	Val30Met	+	+ 32.06	+ 33.22
12	Val30Met	+	+ 32.06	+ 32.07
13	Val30Met	+	+ 32.06	+ 31.77
14	Val30Met	+	+ 32.06	+ 32.45
15	Val30Met	+	+ 32.06	+ 31.33
16	Val30Met	+	+ 32.06	+ 32.46
17	Val30Met	+	+ 32.06	+ 30.75
18	Val30Met	+	+ 32.06	+ 31.29
19	Val30Met	+	+ 32.06	+ 32.73
20	Val30Met	+	+ 32.06	+ 32.57
21	Val30Met	+	+ 32.06	+ 31.22
22	Val30Met	+	+ 32.06	+ 31.54
23	Val30Met	+	+ 32.06	+ 31.35
24	Val30Met	+	+ 32.06	+ 31.82
25	Val30Met	+	+ 32.06	+ 31.72
26	Val30Met	+	+ 32.06	+ 31.30
27	Val28Ser	+	- 12.06	- 13.25
28	Val28Met	+	+ 32.06	+ 32.05
29	Gly47Arg	+	+ 99.13	+ 98.72
30	Thr49Ser	+	- 14.03	- 13.83
31	Thr49Ile	+	+ 12.05	+ 8.79
32	Gly53Glu	+	+ 72.06	+ 71.02
33	Thr60Ala	+	- 30.03	- 29.22
34	Glu61Lys	–	- 0.94	ND
35	Glu61Lys	–	- 0.94	ND
36	Glu61Lys	–	- 0.94	ND
37	Lys80Arg	+	+ 28.01	+ 27.76
38	Gly83Arg	+	+ 99.13	+ 98.77
39	Glu89Gln	–	- 9.90	ND
40	Ala97Gly	+	- 14.02	- 13.55
41	Tyr114Ser	+	- 76.10	- 78.98
42	Tyr114Cys	+	- 60.03	- 59.56

*Abbreviation*: *ND* not detected

### Direct MALDI method to detect variant TTRs in serum samples

**Fig. 1:**
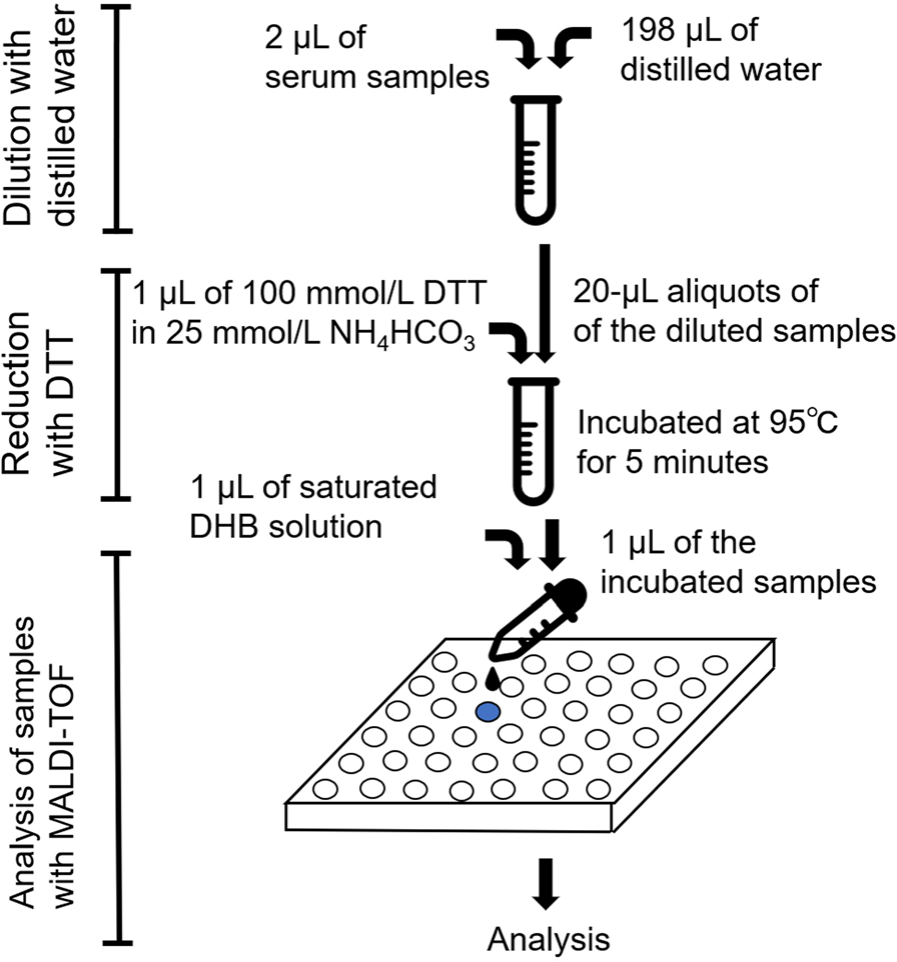
Schematic overview of the direct MALDI method to detect variant TTRs in serum samples. DTT, dithiothreitol; DHB, 2,5-dihydroxybenzoic acid

Serum samples (2 μL each) were diluted 100-fold with distilled water. Aliquots of 20 μL of the diluted samples were mixed with 1 μL of 100 mmol/L dithiothreitol solution in 25 mmol/L NH_4_HCO_3_, and then samples were incubated at 95 °C for 5 min. Aliquots of 1 μL of the incubated samples were mixed with 1 μL of 20 mg/ml 2,5-dihydroxybenzoic acid solution (Bruker, Billerica, MA, USA), and then 1 μL aliquots of the mixed samples were loaded onto the target plate and were air dried at room temperature. The samples were analyzed in a mass range between 1000 and 20,000 Da with MALDI-TOF MS (Autoflex Speed; Bruker) (Fig. 1). The ion peaks were calibrated with insulin (average m/z: 5734.51), ubiquitin I (average m/z: 8565.76), cytochrome c (average m/z: 12360.97) and myoglobin (average m/z: 16952.30) (Protein Calibration Standard I; Bruker).

### Genetic testing

We analyzed the *TTR* genes as previously described [15].

## Results

### Simple and rapid detection of variant TTRs in serum samples from patients with ATTRv amyloidosis by using MALDI-TOF MS without pre-purification

By using a one-step procedure without pre-purification (direct MALDI), within 30 min we successfully detected variant TTR Val30Met in addition to WT TTR in serum samples from ATTRv amyloidosis patients with a heterozygous TTR Val30Met mutation. The direct MALDI method demonstrated that the measured mass shift between variant TTR Val30Met and WT TTR was 32 Da, which was consistent with the theoretical mass shift.

**Fig. 2:**
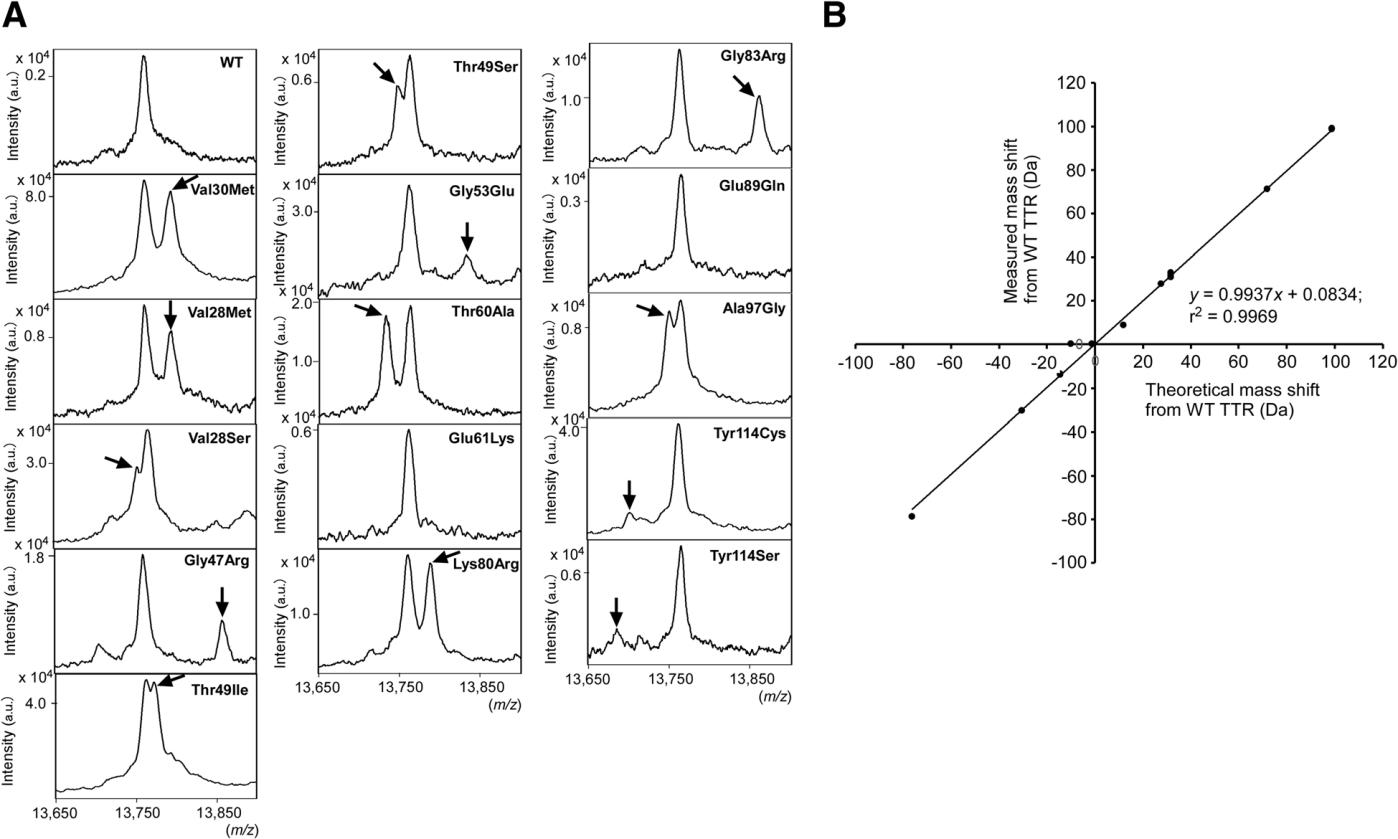
Detection of variant TTRs by using direct MALDI. **a** Mass peaks of TTRs in serum samples obtained by means of direct MALDI. Arrows point to variant TTR peaks. **b** Correlation between measured and theoretical mass shifts (*y* = 0.9937*x* + 0.0834; r^2^ = 0.9969)

We next analyzed 42 serum samples obtained from 42 patients with ATTRv amyloidosis (Table 1). On the basis of genetic analysis of the *TTR* gene, these patients with ATTRv amyloidosis had 15 different TTR mutations: Val30Met, Val28Ser, Val28Met, Gly47Arg, Thr49Ser, Thr49Ile, Gly53Glu, Thr60Ala, Glu61Lys, Lys80Arg, Gly83Arg, Glu89Gln, Ala97Gly, Tyr114Ser, and Tyr114Cys (Table 1). With the direct MALDI method, we detected variant TTRs in 38 (91%) of 42 serum samples (Fig. 2a, Table 1). Direct MALDI could not distinguish between two variant TTRs—Glu61Lys and Glu89Gln. The theoretical mass shift difference between Glu61Lys and Glu89Gln variant TTRs and WT TTR were 0.94 and 0.99 *m/z*, respectively (Table 1), which were thought to be insufficient to separate these variant TTRs from WT TTR by direct MALDI. We detected only WT TTR in all 246 serum samples obtained from 246 patients without TTR mutations by using direct MALDI.

### Correlation between measured and theoretical mass shifts of variant TTRs from WT TTR in serum samples from patients with ATTRv amyloidosis with different TTR mutations

We measured the mass shifts of variant TTRs by the direct MALDI method and compared them with the theoretical mass changes. Direct MALDI revealed a highly significant correlation between measured and theoretical mass shifts from WT TTR in serum samples from ATTRv amyloidosis patients with different TTR mutations (r^2^ = 0.9969; Fig. 2b).

## Discussion

Using this direct MALDI method, we rapidly detected variant TTRs in serum samples obtained from patients with ATTRv amyloidosis. We found a highly significant correlation between measured and theoretical mass shifts from WT TTR in serum samples from these patients.

Development of disease-modifying therapies for ATTRv amyloidosis has made dramatic advances. Liver transplantation (LT) has been performed to halt the progression of ATTRv amyloidosis. LT can lead to mutant TTR synthesized by a diseased liver being replaced with WT TTR produced by the healthy liver graft [16, 17]. In addition, TTR tetramer stabilizers such as tafamidis and diflunisal were developed to prevent dissociation of the TTR tetramer to monomers and to inhibit progression of this disease [18, 19]. Clinical studies have also revealed that gene-silencing therapies, such as the use of small interfering RNA and antisense oligonucleotides that target the *TTR* gene, dramatically reduced disease-causing TTR expression by the liver and significantly improved symptoms of patients with ATTRv amyloidosis [20, 21]. Early diagnosis of ATTRv amyloidosis is becoming more important so that these new disease-modifying therapies may be utilized earlier.

To detect variant TTR in serum samples from patients in a one-step procedure, we developed a new direct MALDI method, which did not require pre-purification such as immunoprecipitation with anti-TTR antibodies. This method required only 30 min to obtain results. Other mass spectrometric methods, such as IP-MALDI, IP-ESI, and SELDI, needed significant time to detect variant TTRs in serum samples because of cumbersome pre-purification such as immunoprecipitation with anti-TTR antibodies or use of the ProteinChip system (Table 2) [7–14]. Direct MALDI is thus a simple means of detecting TTR variants for clinical screening compared with other methods.

**Table 2:** Comparison of different mass spectrometric methods to detect variant TTRs in serum samples

Parameter	IP-ESI [10, 11]	IP-MALDI [7–9]	SELDI-TOF MS [13, 14]	Direct MALDI (the present study)
Immunoprecipitation	+	+	–	–
ProteinChip system	–	–	+	–
Running cost	Low	Low	High	Low
Detection time	2 days	2 days	3 h	30 min
Minimum mass difference that can be identified in each system	14 Da	13 Da	15 Da	12 Da

Our direct MALDI method demonstrated a high sensitivity (91%) for detection of variant TTRs in our patients with various TTR mutations, although it failed to detect the rare TTR variants Glu61Lys and Glu89Gln, whose mass differences from WT TTR were 0.94 and 0.99 *m/z*, respectively. We should therefore note that direct MALDI could not distinguish mass differences of rare variant TTRs with small mass shifts, that is, TTR variants whose mass differences from WT TTR were less than 12 Da (Table 1). Direct MALDI succeeded, however, in detecting most variant TTRs, including the common Val30Met TTR. Direct MALDI may thus be useful for the screening of ATTRv amyloidosis.

Genetic testing is the most reliable tool for diagnosing inherited diseases. However, genetic testing usually takes a long time and is not infallible because of human errors [22]. Therefore, to avoid misdiagnosis of ATTRv amyloidosis, we need an accurate system for double-checking results. Direct MALDI therefore promises to be a valuable tool for double-checking the diagnosis of ATTRv amyloidosis.

## Conclusions

In conclusion, direct MALDI is a simple and quick method to detect serum variant TTRs and is useful for screening of ATTRv amyloidosis.

## Data Availability

The datasets used and/or analyzed during this study are available from the corresponding author upon request.

## Abbreviations

ATTRv: Hereditary transthyretin amyloidosis
ESI: Electrospray ionization
IP: Immunoprecipitated
LT: Liver transplantation
MALDI-TOF MS: Matrix-assisted laser desorption-ionization time-of-flight mass spectrometry; *m/z*, mass-to-charge ratio
SELDI-TOF MS: Surface-enhanced laser desorption/ionization time-of-flight mass spectrometry
TTR: Transthyretin
WT: Wild-type

## References

[1] Ando Y, Coelho T, Berk JL (2013). Guideline of transthyretin-related hereditary amyloidosis for clinicians. Orphanet J Rare Dis.

[2] Mutations in hereditary amyloidosis: mutations in transthyretin gene (*TTR*). http://amyloidosismutations.com/mut-attr.php. Accessed 10 Aug 2018.

[3] Benson MD, Kincaid JC (2007). The molecular biology and clinical features of amyloid neuropathy. Muscle Nerve.

[4] Sekijima Y, Wiseman RL, Matteson J (2005). The biological and chemical basis for tissue-selective amyloid disease. Cell.

[5] Koike H, Misu K, Ikeda S (2002). Type I (transthyretin Met30) familial amyloid polyneuropathy in Japan: early- vs late-onset form. Arch Neurol.

[6] Koike H, Hashimoto R, Tomita M (2011). Diagnosis of sporadic transthyretin Val30Met familial amyloid polyneuropathy: a practical analysis. Amyloid.

[7] Terazaki H, Ando Y, Misumi S (1999). A novel compound heterozygote (FAP ATTR Arg104His/ATTR Val30Met) with high serum transthyretin (TTR) and retinol binding protein (RBP) levels. Biochem Biophys Res Commun.

[8] Tachibana N, Tokuda T, Yoshida K (1999). Usefulness of MALDI/TOF mass spectrometry of immunoprecipitated serum variant transthyretin in the diagnosis of familial amyloid polyneuropathy. Amyloid.

[9] Theberge R, Connors L, Skinner M (1999). Characterization of transthyretin mutants from serum using immunoprecipitation, HPLC/electrospray ionization and matrix-assisted laser desorption/ionization mass spectrometry. Anal Chem.

[10] Ando Y, Ohlsson PI, Suhr O (1996). A new simple and rapid screening method for variant transthyretin-related amyloidosis. Biochem Biophys Res Commun.

[11] Kishikawa M, Nakanishi T, Miyazaki A (1996). Simple detection of abnormal serum transthyretin from patients with familial amyloidotic polyneuropathy by high-performance liquid chromatography/electrospray ionization mass spectrometry using material precipitated with specific antiserum. J Mass Spectrom.

[12] Ranlov I, Ando Y, Ohlsson PI (1997). Rapid screening for amyloid-related variant forms of transthyretin is possible by electrospray ionization mass spectrometry. Eur J Clin Investig.

[13] Ueda M, Misumi Y, Mizuguchi M (2009). SELDI-TOF mass spectrometry evaluation of variant transthyretins for diagnosis and pathogenesis of familial amyloidotic polyneuropathy. Clin Chem.

[14] Tasaki M, Ueda M, Obayashi K (2016). Rapid detection of wild-type and mutated transthyretins. Ann Clin Biochem.

[15] Ueda M, Horibata Y, Shono M (2011). Clinicopathological features of senile systemic amyloidosis: an ante- and post-mortem study. Mod Pathol.

[16] Benson MD (2013). Liver transplantation and transthyretin amyloidosis. Muscle Nerve.

[17] Yamashita T, Ando Y, Okamoto S (2012). Long-term survival after liver transplantation in patients with familial amyloid polyneuropathy. Neurology.

[18] Coelho T, Maria LF, Martins da Silva A (2012). Tafamidis for transthyretin familial amyloid polyneuropathy: a randomized, controlled trial. Neurology.

[19] Berk JL, Suhr OB, Obici L (2013). Repurposing diflunisal for familial amyloid polyneuropathy: a randomized clinical trial. JAMA.

[20] Coelho T, Adams D, Silva A (2013). Safety and efficacy of RNAi therapy for transthyretin amyloidosis. N Engl J Med.

[21] Ackermann EJ, Guo S, Booten S (2012). Clinical development of an antisense therapy for the treatment of transthyretin-associated polyneuropathy. Amyloid.

[22] Shibata Y, Matsushima M, Yabe I (2017). Pseudo-homozygous mutation due to a primer site polymorphism in hereditary ATTR amyloidosis: a pitfall of PCR-based genetic testing. Amyloid.

